# Digitalised multidisciplinary conferences effectively identify and prevent imaging-related medical error in intensive care patients during the COVID-19 pandemic

**DOI:** 10.1038/s41598-024-83978-0

**Published:** 2025-01-07

**Authors:** Gloria Muench, Denis Witham, Kerstin Rubarth, Elke Zimmermann, Susanne Marz, Damaris Praeger, Viktor Wegener, Jens Nee, Marc Dewey, Julian Pohlan

**Affiliations:** 1 Department of Radiology, Charité - Universitätsmedizin Berlin, Corporate Member of Freie Universität Berlin and Humboldt-Universität zu Berlin, Charitéplatz 1, 10117 Berlin, Germany; 2 Department of Cardiology, Charité - Universitätsmedizin Berlin, Corporate Member of Freie Universität Berlin and Humboldt-Universität zu Berlin, Charitéplatz 1, 10117 Berlin, Germany; 3 Institute of Biometry and Clinical Epidemiology, Charité - Universitätsmedizin Berlin, Corporate Member of Freie Universität Berlin and Humboldt-Universität zu Berlin, Charitéplatz 1, 10117 Berlin, Germany; 4 Institute of Medical Informatics, Charité - Universitätsmedizin Berlin, Corporate Member of Freie Universität Berlin and Humboldt-Universität zu Berlin, Charitéplatz 1, 10117 Berlin, Germany; 5 Berlin Institute of Health at Charité - Universitätsmedizin Berlin, Charitéplatz 1, 10117 Berlin, Germany; 6 Department of Surgery with Intensive Care, Charité - Universitätsmedizin Berlin, Corporate Member of Freie Universität Berlin and Humboldt-Universität zu Berlin, Charitéplatz 1, 10117 Berlin, Germany; 7 Department of Anaesthesiology and Operative Intensive Care Medicine, Charité - Universitätsmedizin Berlin, Corporate Member of Freie Universität Berlin and Humboldt-Universität zu Berlin, Charitéplatz 1, Berlin, Germany; 8 Department of Nephrology and Intensive Care, Charité - Universitätsmedizin Berlin, Corporate Member of Freie Universität Berlin, Humboldt Universität zu Berlin, Charitéplatz 1, 10117 Berlin, Germany; 9 Deutsches Herzzentrum der Charité - Medical Heart Center of Charité and German Heart Institute Berlin, Berlin, Germany

**Keywords:** Computed tomography, Radiography, Medical imaging, Magnetic resonance imaging

## Abstract

This study aims to assess the effectiveness of digital multidisciplinary conferences (MDCs) in preventing imaging-related quality management (QM) events during the coronavirus-disease-19 (COVID-19) pandemic. COVID-19 challenged interdisciplinary exchange and QM measures for patient safety. Regular MDCs between radiologists and intensive care unit (ICU) physicians, introduced in our hospital in 2018, enable re-evaluation of imaging examinations and bilateral feedback. MDC protocols from 2020 to 2021 were analysed regarding imaging-related QM events. Epidemiological data on COVID-19 were matched with MDCs. 333 MDCs including 1324 radiological examinations in 857 patients (median age = 64 (IQR = 55–73) years, 66.7% male) were analysed. MDCs were held within a median of 1 day after imaging (IQR = 1–3). QM events were identified in 2.7% (*n* = 36/1324) of examinations. This represented a significant decrease compared to a control group from 2018/2019 (QM events identified in 14.0%, *p* < 0.001). QM incidence remained consistent in the pandemic cohort (regression coefficient estimate = -0.01, 95% confidence interval = [0.000, 0.000], *p* = 0.68). 81% (*n* = 29/36) of QM events were report-related, 19% process-related (*n* = 6/36), and 2.8% indication-related (*n* = 1/36). In 7.3% (*n* = 97/1324) of examinations, the patient was affected by COVID-19. With MDCs as an effective feedback mechanism in place, the challenges of the COVID-19 pandemic led to no increase in QM incidence. Notably, COVID status did not impact QM event occurrence.

**Supplementary Information:**

The online version contains supplementary material available at 10.1038/s41598-024-83978-0.

## Introduction

Imaging is central in the diagnostic workup but remains prone to error^1–3^. Imaging-related medical error, i.e. quality management (QM) events, may affect the indication for an imaging examination, the procedure of image gaining, and image interpretation^4^. Multidisciplinary conferences (MDCs) between radiologists and intensive care physicians held in our institution are associated with a reduction of medical error over time^4^.

The emergence of Severe acute respiratory syndrome 2 (SARS-CoV-2) and coronavirus-disease-19 (COVID-19) led to an enormous burden on healthcare systems around the world^5,6^. Even years after the onset of the pandemic, the variability in the clinical manifestation of COVID-19 still poses diagnostic and therapeutic challenges^7,8^. The radiology department has been central in the diagnostic workup: Imaging plays an important role in the evaluation of COVID-19 lung affection, the differentiation to additional bacterial superinfection and the detection of additional infectious foci^9–13^. Interpretation of COVID-19 chest scans is prone to perception bias and varies inter-individually^14,15^. Thus, multidisciplinary exchange is important in order to benefit from the experience each specialty has been gaining and to reduce the likelihood of medical error.

Interprofessional consultation between radiologists and clinicians has been shown to improve patient care^4,16^. However, the pandemic has fundamentally changed the ways of communication. Severe measures, especially the reduction of face-to-face interaction, were taken by governments to reduce viral transmission^17^. Consequently, direct multidisciplinary exchange has become increasingly difficult when trying to avoid in-person contact. In-person contact was replaced with online meetings, which drastically changes our perception of audio and visual input^18–21^. Thus, an analysis of error detection and prevention in times of digitalised interpersonal exchange is essential.

In hospitals, COVID-positive patients are isolated from non-infected patients^22^ while radiology departments are required to examine both COVID-positive and -negative patients. This side-by-side treatment of infected and non-infected patients is especially resource intensive. Time-consuming protective measures are necessary, e.g., the application of protective gear and special cleaning measures^23^. Intensive care patients, regardless of their COVID status, require special handling when undergoing imaging, e.g., mobile ventilation. Such measures are personnel-intensive, which may further aggravate existing staff shortages^24,25^. Severe stress and exhaustive working conditions in healthcare have aggravated during the COVID-19 pandemic and thus potentially increase the likelihood of error^26–28^.

Thus, measures for error prevention and improvement of multidisciplinary communication became ever more important in the situation of the rising COVID-19 pandemic. In our department MDCs had been established as a quality management (QM) initiative previously. Intensive care physicians and radiologists meet regularly to discuss recent radiological examinations and their first written interpretation. As a main alteration in the context of the COVID-19 pandemic, MDCs were held in an online video format in order to comply with social distancing. This study aims at assessing the effectiveness of MDCs in preventing imaging-related QM events during the COVID-19 pandemic.

## Methods

### QM initiative

MDCs were introduced along with the opening of one medical and one surgical ICU at our hospital in 2018. A detailed description of said MDCs has been published previously^4^. Prior to the pandemic, MDCs were held in person, but were temporarily switched to an online video format with the introduction of social distancing measures. Radiologists and ICU physicians meet in biweekly conferences, separately for each ICU, to discuss imaging examinations of patients under intensive care treatment. MDCs are not scheduled for public holidays. MDCs follow the principles of common diagnostic demonstrations: recent examinations are enrolled in advance by the treating physicians to allow for adequate preparation. The cases selected require clarification for various reasons including inconclusive clinical presentation, complexity of cases, and open questions regarding imaging. Additionally, examinations of newly admitted patients are usually registered. MDCs are chaired by one of two senior radiologists. Patients are introduced by the treating physicians including clinical presentation, suspected diagnosis, current treatment regime, and clinical question regarding the imaging examination(s). Imaging examinations are demonstrated to all participants in the conference and are re-interpreted by the chairing radiologist. Readings and their implications for further diagnostic tests and treatment are then discussed in a multidisciplinary fashion. To our knowledge, MDCs at our hospital differ from many common radiological demonstrations in that standardised protocols are employed for documentation. QM events, i.e., imaging-related errors, are identified and recorded. QM events are defined as not complying with hospital guidelines in the following categories: indication, procedure and reporting. The classification of an incident as a relevant QM event is left to the case-specific interpretation of the chairing radiologists in consultation with all attendees (examples of QM events are given in supplementary Table 1). Both, oral, and written feedback is provided.

### Study population

**Fig. 1:**
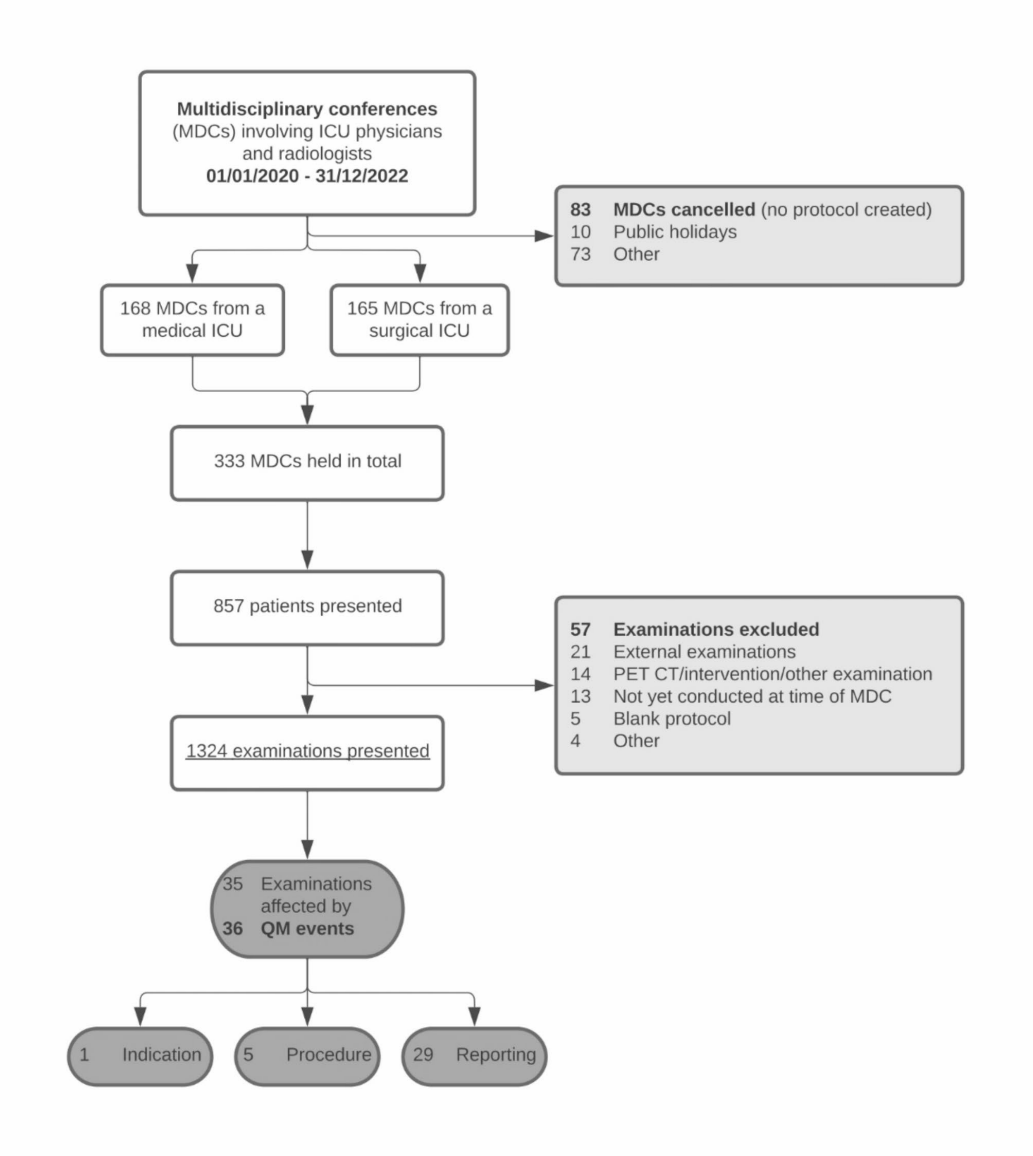
Patient flow chart. Multidisciplinary conferences (MDCs) were introduced for two intensive care units (ICUs) of a university hospital in 2018. These were adapted to an online format with the emergence of the coronavirus-disease-19 (COVID-19) pandemic. Radiologists and ICU physicians meet in biweekly conferences to discuss imaging examinations of mutual patients, identify quality management (QM) events (any imaging-related error), and provide bilateral feedback. A detailed report of such MDCs has been published earlier. Structured protocols of these MDCs were collected over a 2-year period and were analysed with for QM events and COVID status. MDCs were routinely held biweekly, except for public holidays.

We included all ICU patients who were enrolled in an MDC during the period from 1st of January 2020 through 31st of December 2021 (Fig. 1). All scheduled MDCs were included for analysis (*n* = 333). Cancelled MDCs, for which no protocol was created, were excluded. One patient may be enrolled in multiple MDCs with different imaging examinations. Exclusion criteria were external and outpatient imaging examinations. We further categorised all examinations by imaging modality including CT, magnetic resonance imaging (MRI), and X-ray. Other imaging modalities, like sonography or positron emission tomography scans (PET-CTs), and interventions were excluded. Examinations were further categorised by body region examined, i.e., head, chest, abdomen, and other. One examination may be included in several body region categories, if more than one body region was examined in one session (e.g. a combined CT of chest and abdomen was included, both in the chest and abdomen category). Approval of the local ethics committee was gathered under the number EA1/024/21 (Ethikkommission Charité – Universitätsmedizin Berlin). Informed consent has been waived by Ethikkommission Charité – Universitätsmedizin Berlin. The declaration of Helsinki was respected.

### Data collection

This study has a retrospective observational analytic design. Protocols from all consecutive MDCs held during the study period were digitalised using *Microsoft*^®^ *Excel 2016*. Data protection was assured. Further clinical information on all examinations affected by QM events was retrieved from the patient administration system (*SAP* ^®^ *Software 2021 SAP SE or an SAP affiliate company*) and radiological information system (*CentricityTM RIS-I 6 2018 General Electric Company*). Data were collected by a doctoral candidate.

### COVID status

While most COVID-19 patients were treated in separate COVID ICUs newly established in our hospital, the two ICUs included in this study regularly treated both COVID-positive and -negative patients side by side. Relevant COVID affection (i.e., relevant to this specific imaging examination as defined by the ICU physicians) was documented in the MDC protocols and included suspected, confirmed, and recent COVID-19 infection or pneumonia. Relevant COVID affection is referred to as COVID status for the purpose of this manuscript. Proof of a positive COVID status was collected based on positive SARS-CoV-2 PCR and/or typical imaging features on chest CT. Official epidemic data on COVID-19 were gathered from the *Robert Koch Institute (RKI)* website^29,30^. The *RKI* definition of epidemic COVID-19 phases used for this study relies on the following parameters: positivity rate of SARS-CoV-2 PCR testing, PCR tests per 100,000 inhabitants, 7-day-incidence, 7-day-reproduction value, rate of infected individuals with relation to outbreaks, hospitalisation rate, rate of infected individuals with COVID exposure abroad, syndromic surveillance of acute respiratory diseases, COVID-19 rate in ICUs, and current national governmental restrictions.

### Statistical analysis

All statistical analyses were conducted using *Excel*^®^ or *SPSS*^®^. Descriptive statistics are provided as mean and standard deviation (SD) or median and interquartile range (IQR). The QM incidence and time to MDC (i.e., the interval between the imaging examinations itself and the MDC during which it was presented) were compared to a patient population from the same ICUs investigated in 2018–2019 employing Pearson’s Chi-Squared test and Mann-Whitney-U test respectively^4^. Imaging examinations were only discussed once i.e. patients enrolled in multiple MDCs were enrolled with different imaging examinations. Thus, statistical independence was assumed. Due to the low sample size with positive COVID status, no statistical testing was performed to evaluate the difference in QM incidence according to COVID status. The effect of time on the incidence of QM events was calculated using simple linear regression analysis. Epidemic data on COVID-19 and baseline characteristics of MDCs were cross-matched: different epidemic COVID-19 phases were analysed regarding differences in MDC cancellation rate and QM comments using Pearson’s Chi-Squared tests and number of examinations per MDC using ANOVA. The MDC cancellation rate is defined as the percentage of cancelled MDCs, for which no protocol was created, out of all potentially scheduled MDCs. The limit for statistical significance was set at a p-value of 0.05. P-values and confidence intervals were not adjusted for multiplicity due to the explorative study design. Due to the low incidence in the study cohort, no statistical testing was performed for comparison of COVID status and QM incidence between different epidemic COVID-19 phases.

## Results

### Baseline characteristics

A total of 333 MDCs held in an online format were included in this analysis. Of those, 165 were held in a surgical ICU (49.5%) and 168 in a medical ICU (50.5%). During the study period of two years, 1324 radiological examinations of 857 patients were discussed (Fig. 1; Table 1). A median of four (IQR = 3-4.75) examinations was presented per MDC. The median time to MDC was one day (IQR = 1–3). This was shorter compared to a previously published study cohort of the same ICUs (median = 2, IQR = 1–3, *p* = 0.174 ^4^). The most frequently discussed imaging modality was CT (64.0%, *n* = 847/1324), followed by MRI (2.3%, *n* = 30/1324), and X-ray (1.7%, *n* = 22/1324). Body regions examined included the chest (53.9%, *n* = 714/1324), abdomen (28.8%, *n* = 381/1324), head (12.8%, *n* = 169/1324), and pelvis (4.7%, *n* = 62/1324). Imaging was most commonly requested for identification of an infectious focus (34.7%, *n* = 459/1324) including 7.1%, (*n* = 94/1324) examinations with already known or suspected infectious foci, e.g., in patients with pneumonia or pancreatitis. This indication was followed by evaluation of bleeding (8.2%, *n* = 108/1324) and imaging because of acute respiratory distress syndrome (ARDS) (7.2%, *n* = 95/1324).

**Table 1:** Baseline characteristics.

Patients (cases)		Total (*n* = 1279)	Percentage
Sex			
	Male	853	66.7%
	Female Undefined	414 12	32.4% 0.9%
Age	Median (IQR)	64 years (55–73)	
	Male	63 years (53–72)	
	Female	64 years (55–73)	
Examinations		Total (n = 1324)	
MDCs included		333	
Examinations/MDC	Mean (SD)	4.0 (1.3)	
Time to MDC	Median (IQR)	1 day (1–3)	
Imaging modality	CT MRI X-ray	847 30 22	64.0% 2.3% 1.7%
Body region examined	Head Chest Abdomen Pelvis Other	169 714 381 62 41	12.8% 53.9% 28.8% 4.7% 3.1%
COVID status	Positive Negative	97 1227	7.3% 92.7%

Sex and age were calculated on a case basis (defined by an individual hospital identification number, which each patient receives for each new hospital stay), i.e., one patient with several examinations discussed in multidisciplinary conferences (MDCs) during one hospital stay was enrolled only once. If the same patient was re-admitted to the hospital and enrolled in further MDCs, this was counted as another case. In total, 857 patients were enrolled in this study. Age was calculated at the time of MDC. Age could not be calculated in 27 cases. Time to MDC refers to the interval between a radiological examination and its discussion during an MDC. The imaging modality used was not identifiable in 425 examinations. One examination may be included in several body region categories if more than one body region was examined in one session, e.g., a chest/abdomen CT is included both in the chest and abdomen category. The body region examined was not identifiable in 263 patients. The COVID status was calculated on an examination basis, i.e. one patient with several examinations discussed in an MDC may be enrolled in the calculation repeatedly for each examination with relevant COVID-19 affection.

MDC = Multidisciplinary conference.

IQR = Interquartile range.

SD = Standard deviation.

### QM events

A total of 36 QM events were recorded during the study period. Of those, one examination was affected by two QM events. In total, QM incidence was 2.6% (*n* = 35/1324). No repeat errors (i.e., multiple QM events of the same category affecting imaging examinations of the same patient) occurred in this patient population. Twenty-nine QM events affected the radiological report (80.6%), six affected the imaging procedure itself (16.7%), and one the indication for imaging (2.8%). A total of 720 (54.4%) feedback comments were provided in the study protocols, among them 685 for examinations without QM events. For all examinations affected by QM events, written feedback regarding the respective QM event was provided in the standardised MDC protocol. Here, additional aspects of findings or clinical implications were noted, i.e., general feedback was provided to requesting ICU physicians or radiologists involved in the procedure or reporting.

### COVID status and QM events

**Fig. 2:**
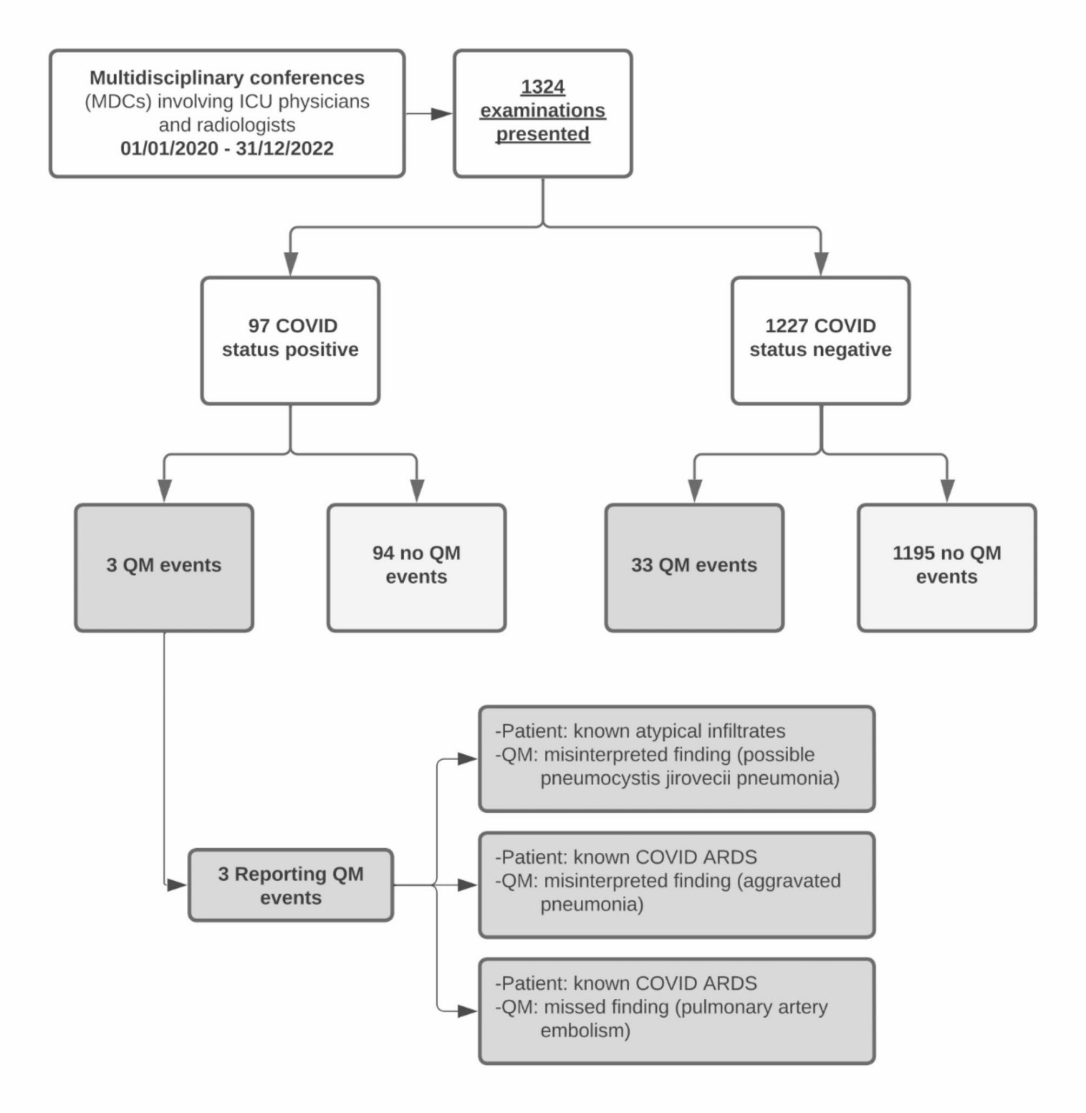
QM events by COVID status. COVID status was noted as documented in the protocol. Relevance of COVID-19 affection is specific to an individual examination and was defined by the experienced ICU physicians involved in MDCs. This included suspected/confirmed/recent COVID-19 infection based on polymerase chain reaction (PCR) testing or computed tomography (CT) imaging. The two ICUs included in this study are specialised in the treatment of medical and surgical conditions respectively. Both ICUs also treated patients with COVID-19 when required for other reasons.

COVID status was positive in 7.3% (*n* = 97/1324) of examinations and in 8.6% (*n* = 3/35) of patients who experienced QM events (Fig. 2). All examinations with positive COVID status discussed in MDCs were CTs. In 44.3% of examinations with positive COVID status, written feedback was provided in the MDC protocol (*n* = 43/97).

### MDCs over time

In a previous study conducted in the same ICUs prior to the COVID-19 pandemic, a QM incidence of 14.0% (*n* = 136/973) was calculated^4^. The QM incidence in the present analysis was significantly lower (2.7%, *n* = 36/1324, *p* < 0.001). The first COVID-19 case in Berlin was confirmed on the 1st of March 2020. The QM incidence remained consistent during the course of the COVID-19 pandemic (Fig. 4). A simple regression analysis yielded no significant change over time (regression coefficient estimate = -0.01, 95% confidence interval = [0.000, 0.000], *p* = 0.68, supplementary Table 2).

### Impact of COVID-19 waves on MDCs

**Fig. 3:**
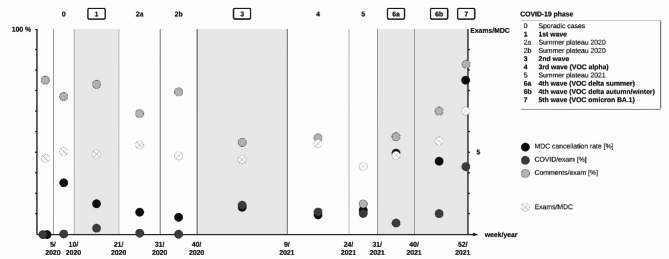
Course of MDCs during the COVID-19 pandemic. MDCs in our hospital were adapted to pandemic requirements with the rise of the COVID-19 pandemic. Radiologist and intensive care physicians met in biweekly QM video calls to discuss mutual patients. The x-axis shows the calendar weeks of 2020 and 2021 separated into different COVID-19 phases as defined by the *Robert Koch Institute* (*RKI*) taking various factors such as incidence, rates of positive PCR-tests, and hospitalisation rate into account^29,30^. COVID-19 waves are highlighted. The left y-axis shows the MDC cancellation rate (out of *n* = 406 potentially scheduled MDCs during the study period) (black), the rate of examinations with relevant COVID affection (as defined by experienced ICU physicians involved in MDCs) (dark gray), and the percentage of examinations discussed in MDCs for which written feedback was provided, i.e., comments/exam (light gray). The right y-axis shows the mean number of examinations that was presented during an MDC for each epidemic COVID-19 phase (supplementary Table 3).

**Fig. 4:**
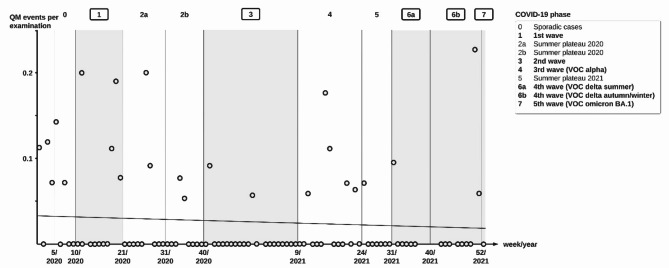
QM incidence over time. The rate of examinations affected by QM events (QM incidence) (y-axis) per MDC is shown for all calendar week (x-axis) of the study period. No decrease in QM incidence was found by simple regression analysis (regression coefficient estimate = -0.01, 95% confidence interval = [0.000, 0.000], *p* = 0.68, supplementary Table 2). Epidemic COVID-19 phases according to the *RKI* are shown^29,30^ and COVID-19 waves are highlighted.

Epidemic COVID-19 phases were defined by the *RKI* incorporating various aspects such as COVID incidence, the rate of positive PCR tests and hospitalisation rates amongst others: first wave, summer plateau 2020 (2a and 2b), second wave, third wave (Variant of concern (VOC) alpha), summer plateau 2021, fourth wave (VOC delta summer and VOC delta autumn/winter) and fifth wave (VOC Omicron BA.1) (Fig. 3)^29,30^. MDC cancellation rate was higher during COVID-19 waves (21.5%, *n* = 58/270) than during low incidence phases (11.0%, *n* = 15/136) (*p* = 0.01). More examinations with positive COVID status were discussed during COVID-19 waves (10.2%, *n* = 87/854) than during low incidence phases (2.1%, *n* = 10/450). The rate of examinations discussed during MDCs for which written feedback comments were provided did not differ between different epidemic COVID-19 phases (*p* = 0.23). The number of examinations discussed per MDC did not differ between epidemic COVID-19 phases (*p* = 0.22). QM incidence remained steady throughout different epidemic COVID-19 phases (Fig. 4).

## Discussion

### Summary

MDCs between ICU physicians and radiologists for identification of imaging-related QM events were introduced in our university hospital in 2018 and initially analysed in an earlier study (2018/2019)^4^. The present study provides follow-up data during the COVID-19 pandemic from 2020 to 2021. MDCs were switched to an online format after the onset of the pandemic. Our results suggest that the rising COVID-19 pandemic had no impact on the incidence of QM events: QM incidence in the present study population remained steady at a consistently lower level compared to our pre-pandemic study population^4^. COVID-positive and -negative patients were equally affected by QM events. The interval between an imaging examination and its discussion during an MDC was shorter in the present study compared to the interval observed in the pre-pandemic study, which started directly after the implementation of MDCs in 2018 ^4^. COVID-19 waves differed from low incidence COVID-19 phases in terms of a higher MDC cancellation rate. Other comparing factors were congruent across different epidemic COVID-19 phases.

### Interpretation

Having established a structured multidisciplinary feedback mechanism between radiologists and ICU physicians in our hospital in 2018, we could rapidly adapt the format to continue early error identification during the COVID-19 pandemic. This was essential for all physicians to gain expertise in coping with the emergence of a new disease. After two years with regular MDCs providing immediate feedback between radiologists and clinicians, the incidence of QM events did not rise despite the tremendous challenges of COVID-19. This may be explained by constant interdisciplinary exchange and a growing feedback culture. During the initial phase of the COVID-19 pandemic, no new radiologists were hired in our department. Thus, the decrease in QM incidence compared to a pre-pandemic study population may be explained by the radiologists’ individual learning curve on the grounds of MDC based feedback. The innovation and implementation of new communication channels during the pandemic facilitated ongoing interpersonal exchange, which appears to be critical for the identification and prevention of medical error. Despite more frequent cancellations of MDCs during COVID-19 waves than during low incidence phases, time to MDC, number of imaging examinations discussed and QM comments provided did not vary. This suggests that the quality of MDCs remained consistent. Such a format seems to provide an effective approach to quality management which may be implemented in other hospitals. Its successful implementation into clinical routine allows for quick adaptation to new situtations, including another rising pandemic. Digitalisation of the format shows to be possible and equally effective.

### Comparison with the literature

The incidence of QM events observed in the present study decreased compared to the incidence we found immediately after the implementation of MDCs in our hospital^4^. This development indicates that MDCs as a QM initiative may be an effective tool for the long-term prevention of imaging-related QM events even in times of a global pandemic. Our data thus suggest that an online format may sufficiently replace in-person meetings when required. To the best of our knowledge, there are no studies analysing the effectiveness of previously established QM interventions during the COVID-19 pandemic. The time to MDC has decreased marginally compared to our initial study^4^. In other words, QM events thus could be identified earlier in the current study, which may have averted potential adverse events. Further characteristics of MDCs have not changed either compared to this initial patient cohort^4^ or during epidemic COVID-19 phases. This indicates a consistently high-quality standard of MDCs, in-person and online, confirming their successful establishment in clinical routine and their effectiveness even during a pandemic. All patients with a positive COVID status in the current study underwent chest CT. Published results show CT to be superior to X-rays in terms of both sensitivity and specificity^10^. This is in line with recommendations of the European Society of Radiology recommending CT as the best imaging modality in the diagnosis of COVID-19 ^22^. Existing studies reported only moderate inter-reader agreement concerning CTs of PCR-positive COVID patients^7^. MDCs aim at reducing the consequences of discrepant reports when imaging studies are interpreted by different readers by contributing to inter-reader consensus through multidisciplinary discussion. Relevant COVID affection reported in this study population was lower compared to the COVID-19 incidence of hospitalised patients^31^. This is owed to the establishment of special COVID ICUs in our institution. Hence, only few COVID patients were treated in the two ICUs included in our analysis.

### Clinical impact

Errors in diagnostic workup may have serious consequences for patients. Early QM identification is therefore of utmost importance. Multidisciplinary exchange is imperative in times of a pandemic but may be especially difficult to realise when all face-to-face interactions must be minimised. Remote multidisciplinary QM conferences may be a suitable approach to keep up a previously established feedback culture. Steps to set up an online format were undertaken immediately and effectively. MDCs were evaluated in a medical and a surgical ICU i.e. they were validated for a wide range of diseases. Thus, the here established format of MDCs may be effectively adjusted to varying challenges in the future. It may also be adopted by other institutions allowing for their adaptation to local requirements. This may continue to improve radiological workup, multidisciplinary cooperation, and ultimately patient safety.

### Limitations

Our retrospective analysis has several limitations. Importantly, causality cannot be established as several confounding factors remain unknown. This study only focussed on imaging-related quality management, an analysis of further modalities and their influence on quality management was beyond the scope of this manuscript and should be the focus of further research. The incidence of both COVID-19 and QM events in the present study is low, limiting the power of the statistical analysis. The retrospective, single-centre design does not guarantee the generalisability of the results to a larger population. However, it ensures consistency in the format of MDCs and the data collection resulting in fewer discrepancies in data quality. As an explorative study, this study merely provides a suitable approach to combat imaging-related error and may be adapted to the hospital-specific circumstances elsewhere. Two senior radiologists led all MDCs, thereby limiting conclusions regarding a larger community of radiologists. Still, the high continuity of the conference format keeps the assessment of QM events constant, allowing further evaluation and identification of relevant factors. The ICUs analysed in the present study did not reflect the level of QM events on primary COVID ICUs that were established in our hospital during the pandemic. This was not within the scope of this manuscript and is the focus of an ongoing study. Our data thus primarily reflect the impact of the COVID-19 pandemic on the management of all patients with common medical and surgical conditions requiring intensive care.

## Conclusion

To conclude, a previously established format of MCDs was adapted to an online format. MDCs continued to identify imaging-related QM events in ICU patients during the COVID-19 pandemic. There was no rise in QM incidence in the face of COVID-related challenges. This format has been shown to be effective, both in a pre-pandemic setting, and during the COVID-19 pandemic. It might be adapted to further epidemic or pandemic challenges that may arise in the future. Further research should focus on digitalised communication channels in quality management.

## Electronic supplementary material

Below is the link to the electronic supplementary material.

Supplementary Material 1

## Data Availability

Data are available from the corresponding author upon reasonable request.

## Abbreviations

ARDS: Acute respiratory distress syndrome
COVID-19: Coronavirus-disease-19
CT: Computed tomography
ICU: Intensive care unit
IQR: Interquartile range
MDCs: Multidisciplinary conferences
MRI: Magnetic resonance imaging
PCR: Polymerase chain reaction
PET CT: Positron emission tomography scan
QM: Quality management
RKI: Robert Koch Institute
SARS-CoV-2: Severe acute respiratory syndrome coronavirus 2
SD: Standard deviation
