# Supplementary material for: Digitalised multidisciplinary conferences effectively identify and prevent imaging-related medical error in intensive care patients during the COVID-19 pandemic
Source: Sci Rep. 2025 Jan 7;15:1197. doi: 10.1038/s41598-024-83978-0 (PMC11706938; doi:10.1038/s41598-024-83978-0)
Supplement: Supplementary file 1 — Supplementary Material 1 [file 41598_2024_83978_MOESM1_ESM.docx]

*Supplementary table 1: Examples of QM events*

|  |  |  |
| --- | --- | --- |
| **Indication** |  |  |
|  | Setting | MRI in patient with necrotic fasciitis |
|  | QM event | Wrong modality requested |
|  | QM comment | “In this case, CT is more suitable than MRI” |
| **Procedure** |  |  |
|  | Setting | Contrast enhanced CT in patient with pyelonephritis and elevated creatinine levels |
|  | QM event | Disregard of contraindications |
|  | QM comment | “Contrast administration despite elevated creatinine levels (4mg/dl)” |
| **Report** |  |  |
|  | Setting | CT in patient under immunosuppression after kidney transplant, atypical pneumonia? |
|  | QM event | Misinterpreted finding |
|  | QM comment | “Possible fungal infection” |

*Supplementary table 2: regression statistics*

|  | R | R^2^ | Std. error of the estimate |  |  | |
| --- | --- | --- | --- | --- | --- | --- |
|  | 0.011 | 0.000 | 0.167 |  |  | |
| **ANOVA** |  |  |  |  |  | |
|  | Sum of squares | df | Mean square | F | P-value F | |
| Regression Residual  Total | 0.005  37.061  37.021 | 1  1322  1323 | 0.05  0.028 | 0.168 | 0.682 | |
| **Coefficients** |  |  |  |  |  | |
|  | Non-standardised coefficients | |  |  | 95% confidence interval for B | |
|  | Coefficient B | Standard error | T | P-value | Lower bounds | Upper bounds |
| **Constant** | 1.501 | 3.595 | 0.418 | 0.676 | -5.551 | 8.553 |
| **MDC** | < 0.001 | 0.000 | -0.410 | 0.682 | 0.000 | 0.000 |

*Supplementary table 3:* *Course of MDCs during the COVID-19 pandemic*

| **Year** | **Week** | **COVID phase RKI** | | **Total MDCs** | **Potential MDCs** | **Examinations** | **QM events** | **QM comments** | **Positive**  **COVID status** |
| --- | --- | --- | --- | --- | --- | --- | --- | --- | --- |
| 2020 | 1-4 | n/a | Not applicable | 14 | 14 | 52 | 4 | 39 | 0 |
| 2020 | 5-9 | 0 | Sporadic cases | 15 | 20 | 61 | 2 | 41 | 0 |
| 2020 | 10-20 | 1 | 1st wave | 34 | 40 | 133 | 8 | 98 | 4 |
| 2020 | 21-30 | 2a | Summer plateau 2020 2a | 34 | 38 | 148 | 2 | 87 | 1 |
| 2020 | 31-39 | 2b | Summer plateau 2020 2b | 33 | 36 | 126 | 2 | 87 | 0 |
| 2020-2021 | 40-8 | 3 | 2nd wave | 76 | 88 | 277 | 2 | 125 | 39 |
| 2021 | 9-23 | 4 | 3rd wave (VOC alpha) | 49 | 54 | 217 | 7 | 103 | 24 |
| 2021 | 24-30 | 5 | Summer plateau 2021 | 25 | 28 | 83 | 1 | 12 | 9 |
| 2021 | 31-39 | 6a | 4th wave (VOC delta summer) | 22 | 36 | 84 | 2 | 40 | 4 |
| 2021 | 40-51 | 6b | 4th wave (VOC delta autumn/winter) | 30 | 48 | 137 | 6 | 83 | 14 |
| 2021 | 52 | 7 | 5th wave (VOC omikron BA.1) | 1 | 4 | 6 | 0 | 5 | 2 |
